# Multi-analyte sensing strategies towards wearable and intelligent devices

**DOI:** 10.1039/d2sc03750e

**Published:** 2022-09-24

**Authors:** Yu Huang, Cihui Luo, Fan Xia, Yanlin Song, Lei Jiang, Fengyu Li

**Affiliations:** State Key Laboratory of Biogeology and Environmental Geology, Engineering Research Center of Nano-Geomaterials of Ministry of Education, Faculty of Material Science and Chemistry, China University of Geosciences Wuhan 430074 P. R. China; Key Laboratory of Green Printing, Institute of Chemistry, Chinese Academy of Sciences Beijing 100190 China; Key Laboratory of Bio-Inspired Smart Interfacial Science and Technology of the Ministry of Education, School of Chemistry and Environment, Beihang University Beijing 100191 China; College of Chemistry and Materials, Jinan University Guangzhou 510632 China lifengyu@jnu.edu.cn

## Abstract

Continuous efforts to produce functional nanomaterials and flexible/stretchable devices have promoted cumbersome, laboratorial, detection processes toward wearable and portable intelligent sensing approaches. Responding to the challenges of the multiple analytes, mixtures, and complex components of practical samples, sensing array and multivariate analysis techniques have a significant advantage in terms of superior analytical capabilities, *i.e.*, they are convenient, rapid, sensitive and have high-throughput for multi-analyte identification in food safety, clinical diagnoses, and environmental monitoring. Besides traditional molecular design and recognition mechanisms, materials with micro/nano structures also contribute to strong signals, sensitive responses, and novel properties. In this review, through a new perspective of signal amplification for responsive discrimination, we summarize progress in developing sensing arrays based on diverse micro/nanomaterials and their integrated devices for multi-analyte discrimination. An overview of strategies for constructing sensing arrays through various micro or nano building blocks, including 0D nanoparticle assembly and modification, 1D nanowires and fibers, 2D graphene and textiles, is schematized. Then, portable and wearable devices integrating colorimetric sensors or flexible electrochemical electrodes with the newest microelectronic units and circuit boards are presented. Meanwhile, the latest artificial intelligence (AI) algorithms are introduced for massive data analysis in complex biological and environmental systems. With future developments in facile and accurate discrimination for multi-analyte research, extended applications will gear up in various fields.

## Abstract 2

Continuous efforts to produce functional nanomaterials and flexible/stretchable devices have promoted cumbersome, laboratorial, detection processes toward wearable and portable intelligent sensing approaches.

## Graphical abstract

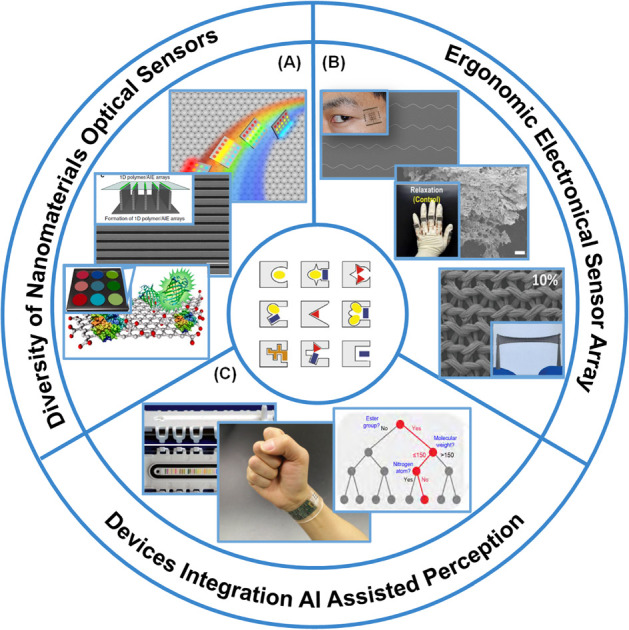

## 1. Introduction

Wearable and intelligent sensing devices have attracted great interest due to their promising prospects in environmental protection and medical health.^1–4^ Their unique advantages in monitoring motion and continuous non-invasive monitoring of dynamic biological fluids, such as tears,^5^ saliva,^6^ and sweat,^7,8^ have also been well demonstrated. Multiple analytes, mixtures, and complex components are the principal features of biological fluids^9–12^ or environmental samples,^13–17^ which provide critical challenges in terms of accuracy, time-consumption, sophisticated instrumentation, and high-cost. Unlike a traditional “lock-and-key” sensor model with highly selective binding interactions to realize a low limit of detection in specific testing, multisensor platforms mimic biological sensory systems (*i.e*., the human nose and tongue) and adopt arrays of broadly cross-reactive sensors to realize “artificial olfactory systems” or an “electronic/chemistry tongue” to discriminate analytes in the gas phase or liquid phase, respectively.^18^ Cross-reactive sensors interact with analytes based on differential binding, and then generate differential response patterns. These response patterns could be considered “finger-prints” of analytes because they are unique for each analyte. Through comparing response patterns, molecular recognition could be successfully realized. Since Persaud *et al.* made models based on a biological olfactory system and adopted different metal oxides as sensors to discriminate similar gases in 1982,^19^ an increasing number of high-throughput sensor arrays have been developed in past decades for multiple analytes, including ions,^20,21^ proteins,^10^ bacteria,^22–24^ and cells.^25,26^ Through changes in chemical or physical properties, such as optical, electrochemical, or electrical, after sensors have combined with analytes, various recognition mechanisms and operating principles have been employed in sensing arrays.^27^

Generally, the biggest challenge in multi-analyte discrimination is the fabrication of multiresolution sensors and the analysis of various and complex data.^28,29^ In traditional sensing arrays, large numbers of sensor compounds or indicators have to be employed to obtain sufficient sensing information, which always involves complicated synthesis or massive screening, which severely limits the development of advanced sensing arrays.^30,31^ Recently, instead of adopting large numbers of sensor compounds or indicators, investigation of micro or nanomaterials has also promoted enormous progress in sensing systems, response mechanisms, testing methods and measurement modes.^32^ Various nanomaterials provide a diversity of morphologies with kaleidoscopic combinations of functional groups and abundant optical or electronical properties. They offer convenient, rapid, sensitive and high-throughput multi-analyte discriminative sensing *via* devices assembled from modified nanomaterials.^33^ Additionally, the integration of devices opens up tremendous opportunities for wild applications based on multi-analyte sensing. In general, sensing arrays provide a large amount of data for processing through statistical analysis, such as principal component analysis (PCA), linear discriminant analysis (LDA), and clustering analyses, *i.e.*, hierarchical cluster analysis (HCA).^34–39^ Briefly, the goal of PCA is to find the greatest extent of variance in a set of data. Linear projection is the critical process for reduction of dimensions and extraction of the main features; that is, reducing the dimensionality of the dataset while identifying and retaining the most important features to describe the original variation. LDA finds a linear combination of features that characterizes or separates multiple analytes, and then adopts distances between centroids to classify analytes. Utilizing the whole dimensionality of the data, HCA creates a dendrogram of clusters to show the similarities between each analyte. Whatever statistical analysis or clustering analyses are adopted to realize successful multi-analyte differential sensing, the key point is to guarantee the sensor array a yield of appreciably differential signals for analysis through array design. A response data matrix is the prerequisite, which contains lots of columns corresponding to the number of detected features, and many rows corresponding to the number of recorded observations.^34^ There are numerous factors contributing to the probability of success in multi-analyte analysis. Deduction suggests a monotonically increasing function for the universality of a strategy applied in multitarget analysis.^40^ The calculated probability of success obviously increases when the number of observations increases. The massive number of different and relevant features will guarantee that the generated signals are cross-reactive and differential. Therefore, features contribute to successfully realizing high-performance multi-analyte discriminative classification and identification. In the response data matrix, an extended or excavated feature column can enrich the data matrix, resulting in an obvious reduction in dimensionality and clear hierarchical linear projection processing. Facing the demands of abundant data for successful multi-analysis, we propose an improvement in multi-analyte recognition *via* response signal and data excavation or extension. Progress in sensing arrays based on the assembly of diverse nanomaterials, the integration of various devices, and the latest developments in data analysis using artificial intelligence (AI) assisted algorithms are summarized to promote multi-analyte identification. A hierarchical outline is expanded with the development of nanomaterial sensors for multi-analysis. A multi-analyte sensor array firstly refers to diversity from nanomaterials to excavate new sensing information or enhance the signal-to-noise ratio. For example, devices containing a novel sensor array are endowed with ergonomic compatibility, multifunctional integration, and deep-learning mathematical operation. Firstly, the diversity of nanomaterials is unfolded in morphology from zero-dimensional (0D) to one-dimensional (1D) and two-dimensional (2D). We describe nanoparticle (NP) assembled nanomatrices for the construction of high-performance optical sensor arrays, and various modifications on NPs for efficient multi-analyte detection.

Optical properties, including structural color, slow-photon fluorescence enhancement, and the plasmon effect, are discussed as features to be excavated to promote more sensitive detection and higher resolution multi-analyte discrimination. Secondly, typical examples of monitoring of multiple vital signs *via* electronic signals are illustrated. The adoption of flexible patterns, e-textiles and graphene gels, and the fascinating prospects for clinical diagnosis and health assessment *via* extending multi-analysis features using piezo-resistance, field effect transistors, and capacitance monitoring are described. Finally, examples of practical devices with the rational integration of materials, structures, and mechanisms to approach portable, wearable, or attachable multi-analysis sensors are given. Beyond the design of devices, integration also involves the advanced mathematic operation of machine-learning. Facing up to a complex realistic environment or the requirements of dynamic monitoring calls for sensitive, high-throughput, commercial, and versatile multi-analyte recognition instruments with good user experience. Nanomaterial-based sensor devices present various reinforced optical or electronical properties *via* morphological construction. The extended and excavated response features will contribute to a tremendous database and result in high-resolution discrimination or abundant multi-analyte recognition.

**Fig. 1:**
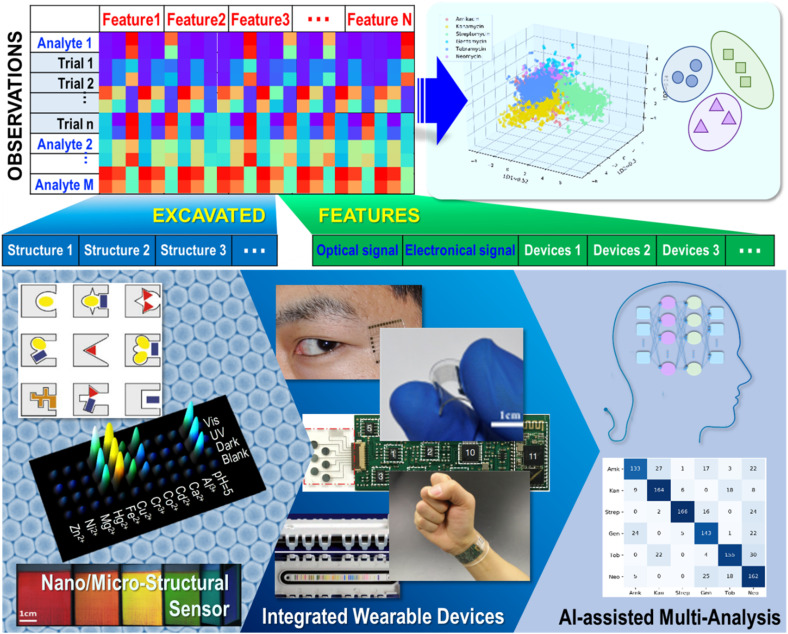
Schematic of promoted multi-analyte discriminative sensing *via* structural diversity, wearable device integration, and artificial intelligence (AI) assisted strategies. Reproduced with permission from ref. 17_,_^28^, ^40^, ^68^, ^73^, ^117^, ^128^ and 129. Copyright 2001, Wiley-VCH. Copyright 2016, Nature Publishing Group. Copyright 2015, Nature Publishing Group. Copyright 2016, Wiley-VCH. Copyright 2015, ACS publishing group. Copyright 2021, Wiley-VCH. Copyright 2022, ACS publishing group.

In this review, an overview of strategies for constructing sensing arrays through various micro or nano building blocks, including 0D NP assembly and modification, 1D nanowires and fibers, and 2D graphene and textiles are presented. Then, typical sensing array devices integrated through chemometric methods for the differential sensing of clinical samples are displayed. Additionally, recent developments in AI-assisted algorithm data analysis over the last two or three years are summarized (Fig. 1). Finally, perspectives on challenges and future developments are presented. With the development of facile and accurate discrimination for multi-analysis research, extended applications would gear up in various fields.

### 2. Diversity of nanomaterial optical sensors

Most recently, tremendous efforts have been made with low-dimensional nanomaterials as highly sensitive sensor transducers, including NPs, nanowires, nanotextiles, nanotubes, nanofibers, and graphene. For fluorescent sensing arrays, the rational design of a series of molecular sensors with similar chemical formulae is the most typical method. In general, these series of sensors are derivatives of a substance, which usually come from chemical modification, such as substitution with extended conjugated fluorescence.^41–45^ However, the rational design of sensors involves complex synthesis, purification processing and compound screening to achieve serials for valid sensors. Now, the field of multi-analyte molecular recognition has reached a stage where one can develop new principles and techniques to design and synthesize a feasible receptor with a good degree of predictability and selectivity. In particular, based on molecular design, improvements in methods and integration of devices, it is crucial to achieve correlative differential analysis without complicated chemical synthesis or screening of valid compounds. Through analyzing the characteristics of the sensor (*i.e.*, optical, photo luminescent, and fluorescent) before and after binding with targets, a serial of targets with similar molecular structures, chemical or physiologic properties (*i.e.*, proteins, anions, or human mobility) can be distinguished. Meanwhile, a series of new issues appear in the multi-analyte analyzing process from an analogical perspective. With the development of nanotechnology, new micro/nanostructure processing techniques have been combined with multi-analyte discrimination to avoid complex rational molecular design, which has obviously geared up their applications in related device integration, such as in biological detection and the electronics industry. In this section, sensing array assemblies and modifications through various micro/nano building blocks, including 0D NPs, 1D nanowires and fibers, 2D graphene and textiles, and their integrated devices for bio-target discrimination are discussed.

### 2.1 NP-assembled photonic crystal (PC) sensing arrays

As the most basic nanomaterial element, 0D NPs have undergone mass exploration since nanomaterial and nanotechnology research began to boom. Various kinds of NPs have been fabricated, diversely coordinated, and assembled with various novel functions. Specifically, through a facile and cost-effective bottom-up self-assembly strategy, photonic crystals (PCs) with a periodic dielectric structure have received increasing interest.^46–52^ PCs are capable of affecting light propagation and create a forbidden gap (a photonic stopband) in their photonic band structure.^53^ In past decades, PCs have attracted enormous research interest and increasing awareness of their potential applications in sensing, detection, catalysts, displays, solar cells and other fields, owing to their specific optical properties. Through reducing the group velocity at the edge of the photonic stopband of PCs, PCs have demonstrated outstanding modulation of spontaneous emission. Therefore, on photonic stopband matched PCs, the light output shows effective modulation and optical signal selective amplification. For PCs fabricated from self-assembled NPs, their photonic stopband can be easily tuned through NP sizes, chemical constitutions, assembly structures, and light incident angles, according to Bragg's law:^54^*λ*_max_ = 2*d*_111_*n*_eff_ sin *θ*Here, *λ*_max_ is the maximum wavelength of reflectance, also called the photonic stopband; *d*_111_ refers to lattice spacing, which is related to size of the NPs and their assembly structure; *n*_eff_ is the effective refractive index of the PCs, decided by the materials of the NPs and the materials between the NPs; and *θ* is the glancing angle. The photonic stopband of the PCs was decided by the materials and size of the NPs, their assembly structure, the light incident angle, and the PCs' functionalized molecular recognition agents during the sensing process. With a matched photonic stopband, PCs would transduce diverse external stimulations to measurable optical responses and selectively enhanced fluorescence signals. Based on this principle, PC-based sensors with high efficiency and selectivity have been well developed, including analytes of metal ions, cations, pH, humidity, oil, DNA, cells, antibodies, glucose, creatinine, and ethanol.^55–58^

Inspired by PC-based sensors, researchers figured out that PCs could provide an opportunity for efficient and facile multi-analyte detection based on the achievement of increased sensing information Cunningham *et al.* extended the DNA detection capabilities of a standard microarray test on PCs by enhancing the fluorescence intensity from microarray spots.^59,60^ Another compelling advantage of a PC-based sensing array in multi-analyte recognition is avoiding a large number of lab-synthesized chemical compounds adopted as sensors. By introducing 6 commercial dyes into 7 PCs with different stopbands, Gu's group developed a PC sensor array for the sensing of 8 vapor-phase chemical molecules.^61^

When the photonic stopband of PCs is in the visible light region, the changes in the photonic stopband are actually displayed as a change in structural color, which can be perceived by the human eye. For example, Li *et al.* combined ionic liquids and appropriate PCs to realize anion detection by the naked eye.^62^ When sensing different anions, counter anion exchange led to changes in the PCs' periodic structure. As a result, the film's color varied according to the different anions. Unlike a traditional PC-based sensor adopting signals in the equilibrium state, Ge and co-workers developed a PC gel to distinguish solvents with similar structures through monitoring its dynamic reflection spectrum.^63^ Similarly, Ozin *et al.* fabricated a “PC nose” through a PC sensor with multiple layers of alternating refractive index.^64^ Nine kinds of various functionalized PCs were adopted to realize identification of similar molecules and bacteria. PCs can also be flexibly combined with other sensing materials: for example, molecularly imprinted polymers. Combining molecularly imprinted and inverse PCs as sensing elements in a sensing array, identification of 6 analytes was reported by Li's group for the first time.^65^ Compared with chromatographic methods, molecularly-imprinted spatially patterned PC arrays demonstrated a significant advance in the low-cost fabrication of sensors, a simple and fast analytical process, and the ability to detect trace amounts.

Although multi-stopband PC chips combined with a serial of chemical sensors have demonstrated their ability to discriminate multiple analytes, researchers are still working on simplifying the sensing array to a mono-chemical sensor or mono-stopband PC chips. In these cases, the complicated synthesis and screening process of serial sensors, and the complex fabrication and integration process of a multiplex PC materials matrix can be avoided. We constructed a multi-stopband PC microchip for the recognition of 12 metal ions based on only one simple and commercialized sensor: 8-hydroxyquinoline (8-HQ).^66^ This PC microchip was fabricated through a hydrophilic–hydrophobic patterned surface. Due to the different surface energies of the hydrophilic area and hydrophobic area, NPs would selectively adsorb and self-assemble on the hydrophilic areas of the patterned surface.^67^ The multi-stopband PC microchip selectively performed fluorescence signal enhancement through the matched photonic stopbands, which contributes to the correlative differential analysis. Instead of adopting 6 kinds of 8-HQ derivatives as sensors to achieve enough differential responsive signal for discriminating metal ions, this work used PCs to amplify the responsive signal after metal ions had combined with 8-HQ, which also successfully realized metal ion recognition. This research provides significant insight into high-performance discriminant analysis through introducing micro/nanodevices.

**Fig. 2:**
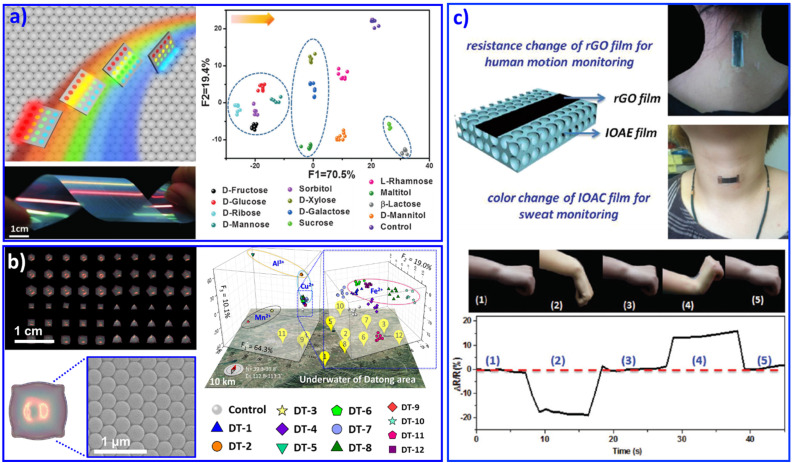
(a) A rainbow structural-color mono PC chip selectively enhances fluorescence with various viewing angles, resulting in the identification of 12 saccharides. Reproduced with permission from ref. ^68^. Copyright 2016, Wiley-VCH. (b) The diverse micromorphology of a PC chip realized the discrimination of various metal ions and 12 groundwater samples. Reproduced with permission from ref. ^69^. Copyright 2021, Wiley-VCH. (c) Multifunctional wearable sensor based on a composite of rGO and PCs. The wearable sensor attached on the wrist joint and neck. Reproduced with permission from ref. ^71^. Copyright 2018, Royal Society of Chemistry.

Furthermore, we developed a rainbow structural-color mono-stopband PC chip assembled from single-size NPs. This PC chip displayed angle-dependent structural colors from red and orange to bluish violet, as shown in Fig. 2a.^68^ According to Bragg's law, besides the lattice spacing and refractive index of the PCs, the angle of incidence of light (*θ*) also plays a key role in changing the photonic stopband position of PCs : PCs displayed various colors when *θ* changed from 0° to 90°. The change in the color implied a shift in the position of the photonic stopband of the PCs, that is, the maximum wavelength of reflectance. The detection process was carried out under various reflection angles, endowing this PC mono-stopband chip with the ability to enhance the fluorescence in different spectral regions. As a result, this mono-stopband PC chip realized the recognition of 12 saccharides through PCs assembled from one size of NPs. Based on this understanding, recently we further developed PC chips with diverse micromorphology assembled from one size of NPs, as shown in Fig. 2b.^69^ The PC pixels displayed various structural colors and provided abundant differential fluorescence information. As a result, 14 metal ions and 12 groundwater samples were successfully distinguished.

Similar to opal PC, inverse opal PC can also be adopted for sensing systems. Combined with poly(ionic liquids) as a sensing unit, the imidazolium moieties generated various products by a counterion exchange reaction of ionic liquids, resulting in the discrimination of 5 anions and 8 organic solvents with different polarities. Poly(ionic liquid) inverse opal PC beads doped with AIE dye were exploited for the recognition of 20 natural amino acids in human urine.^70^ Combined with a flexible substrate, PC-structured wearable sensors with the abilities of *in situ* monitoring of motion and sweat were feasibly achieved. For example, Gu's group developed a multifunctional wearable sensor based on a reduced oxide graphene (rGO) film on a PC film, as shown in Fig. 2c. The wearable sensor can be attached on the wrist joint and the neck to realize *in situ* motion monitoring and *in situ* analysis of the ion concentration in sweat.^71^ Besides polymer NPs, metal NPs could also be adopted to form micro/nanostructural sensor arrays.^72^ We assembled Ag NPs on flexible poly(dimethylsiloxane) (PDMS) with a patterned silicon pillar to integrate curve arrays.^73^ The as-prepared Ag NP curve arrays displayed discriminative resistance with different deformations, which endowed it as a wearable microelectrode sensor for discriminating monitored human facial expressions, including relaxed, anger, disgust, fear, laughter, sadness, smiling, and surprise. We believe that with burgeoning inkjet printing technology, the design and fabrication of patterned PC-based sensing arrays for the recognition of various multiple analytes will become a remarkable part of wearable sensing chips, due to their advantages of efficient use of materials, low cost, and elimination of waste. This idea may open an efficient avenue for the multi-analyte recognition research field.

### 2.2 Nanofiber aligned sensor array

**Fig. 3:**
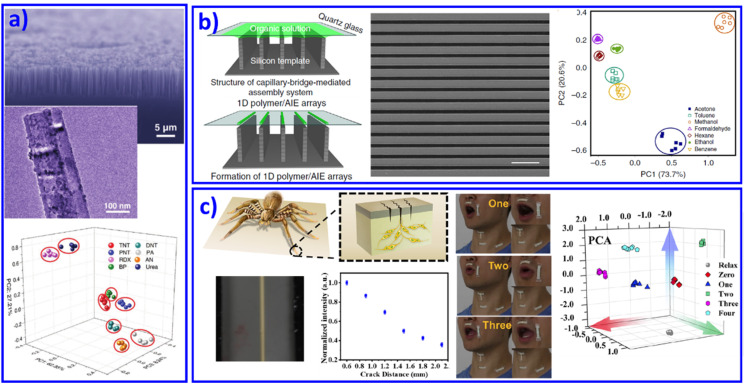
1D nanomaterial based sensor arrays. (a) A SiNWs/ZnO/rGO Schottky sensor array for vapor recognition. Reproduced with permission from ref. ^77^. Copyright 2017, Wiley-VCH. (b) Organic vapor discrimination. Reproduced with permission from ref. ^79^. Copyright 2018, Nature Publishing Group. (c) Inspired by slit in spider's leg joint, ultrasensitive mechanoluminescence sensors were achieved for visualizable vocalization. REF Reproduced with permission from ref. ^80^. Copyright 2021, American Chemical Society.

Over past decades, 1D nanoscale building blocks with superior electrical and mechanical properties, such as nanowires, nanofibers, and nanotubes, have emerged as key components in a number of advanced and miniaturized electronic, sensing, optical, electromechanical and thermal systems. Meanwhile, because of their high surface-to-volume ratios, 1D materials are considered particularly sensitive chemical sensors. Introducing 1D nanomaterials as different active materials into array sensing devices has also attracted immense interest. For instance, Heath *et al.* and Li *et al.* developed functionalized Si nanowires (NWs)^74^ and carbon nanotubes^75,76^ as sensing elements in sensor arrays to distinguish vapors. Most recently, Dou *et al.* designed and fabricated an optoelectronic SiNWs/ZnO/reduced graphene oxide (rGO) Schottky sensing system for the recognition of explosive vapors.^77^ The ultrasensitive light response property of SiNWs and the response to explosive vapors of SiNWs and ZnO NPs contributed to a real-time sensing array, as shown in Fig. 3a.

Although metal and non-metal nanowire based sensing arrays have been demonstrated, challenges still remain in their effective fabrication at large scale. For polymer nanowires, the approaches are much more flexible, providing a significant perspective insight for advanced 1D material based sensing chips. Typical examples include the development of fluorescent sensors combined with a polymer nanofiber mat to achieve sensing of 10 metal ions.^78^ Through an electro-spun method, the fiber's chemical components and orientations in the mat can be easily tuned, demonstrating its potential for various target analytes, even as wearable sensors. Recently, AIE molecules combined with polymer microwires were further developed to form an organic vapor sensing array (Fig. 3b).^79^ The polymer microwires came from a liquid-bridge-mediated assembly method. With organic vapor present, the AIE/polymer microwires would swell and change the aggregated state of the AIE molecules, resulting in varying fluorescent intensity. This new mechanism of swelling-induced varying fluorescent signals provided a new method for optical organic vapor sensing arrays. With this understanding, very recently we mimicked joint slits in spider legs and developed ultrasensitive mechanoluminescence sensors. Under the driving of 0.1 N stress, these sensors with a slit-based pattern performed distinct mechanoluminescence, which contributed to bright luminescence while people talked and during visible vocalization (Fig. 3c).^80^ Further improvement in the performance of the 1D nanomaterial based sensing array, such as selectivity and sensitivity, will generate a significant practical impact on defense applications, environmental monitoring, and medical diagnosis. However, device fabrication and system integration processes still require significant development for economically viable applications.

### 2.3 Graphene-based 2D nanosheet matrix

**Fig. 4:**
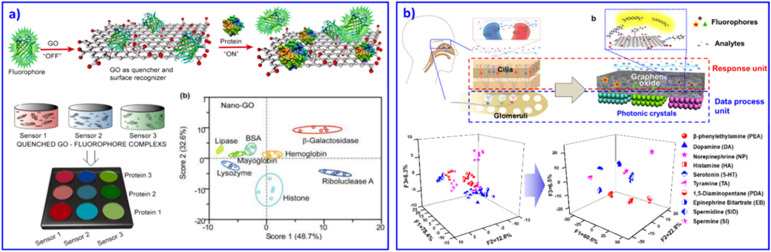
2D nanomaterial-based sensor arrays. (a) GO as receptors for biomolecular interactions and sensing. Fluorescent reporters were initially quenched through GO binding. Displacement of quenched fluorophore by analyte proteins would restore the fluorescence. Therefore, GO–protein interactions can be analysed and discriminated through differential fluorescent change. Reproduced with permission from ref. ^82^. Copyright 2012, American Chemical Society. (b) An olfactory system inspired sensor chip based on a GO and PC bilayer structure sensing system. The GO layer effectively captures the analytes, and PCs selectively enhance fluorescence signals, contributing to multi-amine recognition. Reproduced with permission from ref. ^87^. Copyright 2018, American Chemical Society.

Among various 2D materials, graphene is absolutely a rapidly rising star on the horizon of condensed-matter physics and materials science, with its outstanding properties of good electrical conductivity, high mobility, surface area, high Young's modulus, optical transmittance and so on.^81^ As a precursor, graphite oxide (GO) has emerged, offering the cost-effective, large-scale production of graphene-based materials. For discriminative sensing, GO usually works as a quenching substrate or signal transducer for detecting proteins,^10,82^ therapeutic antibodies,^83^ and distinguishing differences in human mobility^84^ and human hemoglobin.^85^ For example, Fan *et al.* combined nanographene oxide (NGO) and 7 kinds of DNA tagged with fluorescent dye to form a sensing array system.^10^ The fluorescence of the system was quenched at first. With various proteins, the bound DNA strands were released into solution due to competitive displacement of the proteins. As a result, differential fluorescent response patterns were achieved. In this work, they demonstrated that the size and shape of NGO may result in various fluorescent quenching effects. Later on, based on NGO and fluorophores combined into a sensor array, Dravid and co-workers confirmed that the fluorescent response was enhanced as the size of the NGO became smaller, due to a more pronounced GO–protein binding and release response through fluorescence displacement transduction, as shown in Fig. 4a.^82^

Besides GO working as a quencher and changing the fluorescent signal in a sensing array, the conductivity property of GO also contributes to discriminating analytes. Based on inkjet printing, our group developed a wearable graphene aerogel sensing array for deformation responses.^86^ Through the reversible mechanical deformation induced electrical conductivity change of the wearable device, discrimination of complicated movement perceptions could be achieved. Inspired by the olfactory system, we mimicked the cilia and glomeruli to develop a GO and PC bilayer structural sensing chip to discriminate 7 drug amines.^87^ As shown in Fig. 4b, the GO layer captured the analytes and generated fluorescent signals based on its microporous structure. Then, PCs selectively enhanced the fluorescent signals through their periodic structure. As a result, the bilayer integrated sensing chip successfully realized the recognition of similar drug amines. Other 2D materials, such as MnO_2_,^88^ MoS_2_, textiles,^84^ and WS_2_ nanosheets^89^ were also adopted to construct sensor arrays. For example, our group developed a wearable electronic textile for human motion recognition based on commercially available polyester fabrics and silver precursors.^84^ Compared with GO-based devices, the polymer fabric-based devices displayed better deformation performance and much simpler fabrication by just dipping the commercial polyester fabrics into the silver precursor. This strain–electric responsive sensor displayed outstanding sensitivity, durability, and permeability, benefiting its promising applications as wearable devices for sports training, health care monitoring and so on. Based on 2D materials as blocks, the further development of comfortable wearable devices for sensing human health is strongly desired.

## 3. Ergonomic electronic sensor array

Current detecting devices and analysis methods for complex systems are stuck because of the bulky instruments, limited or simple response function, and complex analysis operation. The tremendous desire in clinical diagnosis and health assessment for the monitoring of vital signs calls for high-resolution discrimination and a sensitive response with good user experience. Wearable electronics is fascinating because of its advantages of convenience and non-invasiveness, which combines ergonomic contents with flexible and bio-friendly materials, multi-integration, bionic design, and a smooth human–machine interface. In the following, we will present some examples of flexible patterns, electronic textiles and graphene combinations for human symptoms or monitoring of signs. Static and dynamic basic data of body movement, and changes in humidity and temperature were detected and analyzed for earlier medical diagnosis, long-term chronic monitoring, auxiliary apparatus for people with handicaps or paraplegia, daily health assessment and so on.

### 3.1 Flexible patterns

**Fig. 5:**
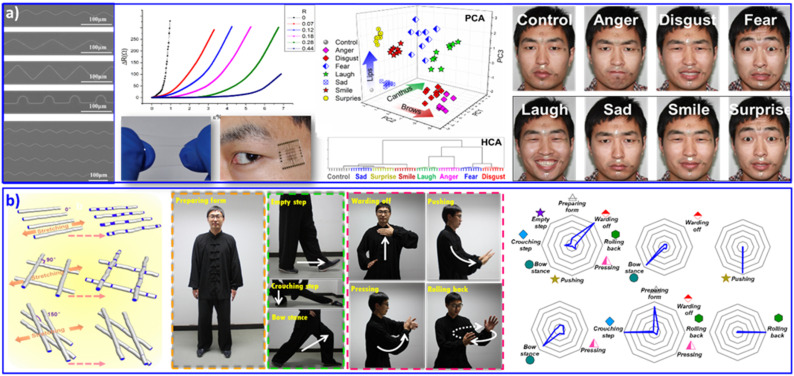
(a) Wearable sensing device based on silver NP assembled curved arrays, with increased electrical resistance in response to strain deformation. The wearable sensing devices were attached at six selective positions on facial skin, contributing to discriminant analysis of eight facial expressions. Reproduced with permission from ref. ^73^. Copyright 2016, Wiley-VCH. (b) Wearable sensing device based on a microfiber-knitted sensor array. The wearable devices were attached at the shoulders, elbows, and knees with different cross-weave angles, contributing to whole-body movement monitoring and multifactor quantitative analysis. Reproduced with permission from ref. ^94^. Copyright 2018, Wiley-VCH.

Flexible electronics,^90,91^ dispersing conductive or semiconductive nanomaterials into elastomeric matrices,^92^ is blooming with the tremendous and increasing demand for integrated circuits, wearable devices and attached or embeddable intelligent instruments. Fractal and serpentine patterns are commonly adopted to separate and release stress during stretching, bending or twisting flexible operations. Generally, in order to achieve a sensitive and accurate elastomeric deformation response, flexible electronics are required to adopt arbitrary curve patterns and tunable tortuous morphologies. As shown in Fig. 5a, we assembled silver NPs into curved arrays with various curvatures *via* pillar-patterned template printing.^73^ The curves with various tortuous morphologies have differential resistive strain sensitivities, which exhibit sensitive and stable resistance responses to deformation. With multichannel monitoring of the delicate deformation of human facial expression, 6 positions on facial skin were selected. These 6 positions contained the basic muscle groups for most expressive movements. Finally, 8 main expressions (anger, disgust, fear, laughter, sadness, smiling, surprise, and a relaxed one as a control) were analyzed. Originating from the distinctive changes in resistance during facial expression, PCA and HCA statistical operations showed complete and clear classification of all 8 monitored expressions. The sensitive perception of skin movement with facial expression recognition contributes to remarkable applications in auxiliary apparatus for skin micromotion manipulation, especially for people with paraplegia.

### 3.2 Electronic textiles

For modern detection and analysis technology, experience is recognized and acknowledged as a critical pursuit. Successful wearable devices will face burgeoning requirements in terms of multifunctionality, adaptability to complex environments and convenience of setup. Textiles, as the most successful ergonomic materials, contribute light weight, permeability, and durable features for human comfort. Textile knitting endows inherent flexibility with diverse knitting morphology to achieve integrated wearable electronic sensing instruments.^93^ As shown in Fig. 5b, we developed arrays of conductive microfibers and then knitted them with various cross-weave patterns to construct multiresolution flexible electronical sensors.^94^ The cross-weave angles of fibers knitted in various patterns exhibit tunable resistive strain sensitivity. Then, sensors with different cross-weave patterns were integrated into multiresolution wearable sensors for whole-body joint kinematic monitoring. Six microfiber-knitted sensors were attached to the human shoulder, elbow and knee joints to monitor shadowboxing movements. Another example is to construct an e-textile sensor on a commercial polyester textile *via* a simple and effective dyeing method. Low-viscosity and low-surface-tension ink with reactive silver ions ([Ag(NH_3_)_2_]^+^[C_2_H_3_O_2_]^−^) was adopted in this case.^95^ The stable electronic dyeing and various textile knitting morphologies respond to specific differentials of imperceptible movements. Therefore, multi-analyte investigation results in delicate distinctions for the experimenter undertaking the same action. E-textiles with multipoint sensing and complicated data analysis could precisely perceive or distinguish the complexity and similarity of human motions, displaying potential applications in movement-related health status and clinical practice.

### 3.3 Graphene combinations

Graphene, as an excellent 2D material that combines many advantages, such as conductivity,^96^ thermal conductivity^97^ and flexibility,^98^ can be easily processed into different morphological nanostructures, including ultrathin films,^99^ nanosheets,^100^ paper,^101^ fiber,^102^ nanoribbons,^103^ foams^104^ and sponges.^105^ The response diversity and mechanical flexibility of graphene materials has contributed to flexible sensitive sensors for collecting a large amount of valid data.^106^ Meanwhile, qualitative or quantitative ergonomic measurement results were also obtained through multivariate analysis. For example, Ryhanen *et al.* designed an ultra-fast graphene film as a human body humidity and temperature sensor.^107^

**Fig. 6:**
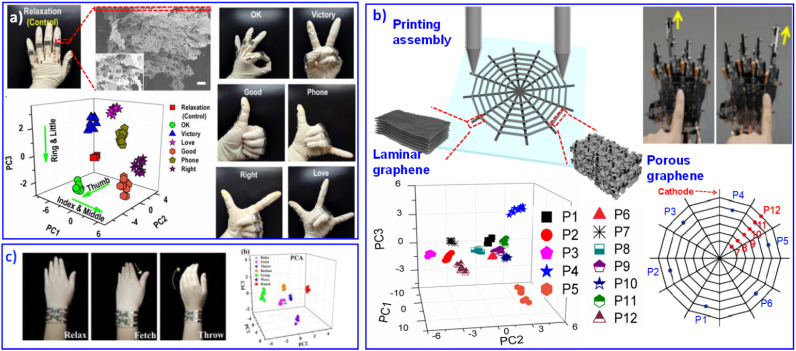
Flexible sensors based on graphene-combination materials. (a) Printed rGO aerogel sensor with multidimensional motion response for gesture recognition. Reproduced with permission from ref. ^86^. Copyright 2016, Royal Society of Chemistry. (b) Touch-sensitive rGO skin with different microstructures to balance stimulated response and signal transmission. Reproduced with permission from ref. ^108^. Copyright 2018, Royal Society of Chemistry. (c) Graphene aerogels with porous serpentine patterns for recognizing wrist movements. Reproduced with permission from ref. ^109^. Copyright 2019, Royal Society of Chemistry.

Through PCA analysis, different whistled songs can also be qualitatively identified. Based on 3D microstructures of graphene aerogel, we printed GO aerogel patterns to monitor human motions.^86^ The 3D architectures of reduced graphene oxide (rGO) can respond to complex mechanical stimulations and achieve multi-gesture recognition (Fig. 6a). Although graphene aerogel contributes to a sensitive flexible response, its performance is still a big obstacle because of its high resistance. Porous structures will result in poor electron transfer between graphene sheets, and the laminar assembled graphene will bring about much higher electron transfer efficiency. Various assembly structures exhibit different electrical behavior with unitary graphene material. Their improved progress combines laminar graphene and aerogel graphene in an integrated sensing device. The porous-structured graphene pattern sensitively responds to micromovements, and the laminar graphene pattern behaves as a good conductor to transmit the sensing signal smoothly. Touch-sensitive graphene skin can perform clear multiresolution orientation, locational kinesis tracking and tactile-sensing control (Fig. 6b).^108^ Recently, we developed graphene aerogels with porous serpentine patterns, displaying various multidimensional deformation responses.^109^ Then, wrist movements can be successfully recognized, as shown in Fig. 6c. The graphene skin developed by our group showed promising adoptability in the future multifunctional integration of electronics.^109^ The introduction of multi-analysis technology enables efficient discrimination of various and complex data. Multi-analyte detection of complex environments enables sensor components with high sensitivity, fast response, and low detection limits for high-resolution signal reception and efficient transmission.^110^

## 4. Device integration

In previous sections, we discussed nanomaterial-based multi-analyte bio/chemical sensing arrays, as well as design and assembly strategies for wearable electronics with unique electrical and mechanical properties for the monitoring of multiple vital signs. Additionally, the integration of circuits and systems opens up tremendous opportunities for multitarget sensing applications spanning bio/chemical sensing, stimulation, to clinical pathology, diagnosis and therapy. In this section, examples of such integrated systems in the forms of hand-held devices and multifunctional skin are reviewed, and the latest developments in AI-assisted data analysis methods are also discussed.

### 4.1 Hand-held devices

**Fig. 7:**
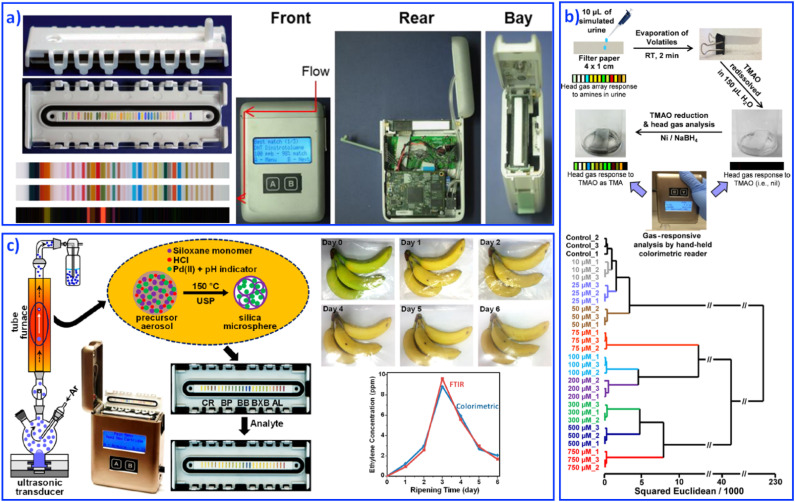
Hand-held devices containing colorimetric sensor arrays. (a) Structure of hand-held devices. Reproduced with permission from ref. ^117^. Copyright 2015, American Chemical Society. (b) Quantification of urinary trimethylamine N-oxide. Reproduced with permission from ref. ^118^. Copyright 2018, American Chemical Society. (c) Monitoring CO and ethylene. Reproduced with permission from ref. ^119^. Copyright 2019, American Chemical Society.

The development of fundamental research into sensing array systems calls for the generation of user-friendly, highly sensitive, inexpensive and disposable sensing devices. Various materials, such as paper-based,^111^ organic field effect based,^112^ and gel based^113^ sensing arrays were developed as integrated sensing devices for reliable qualitative and quantitative analyses. However, the above-mentioned instruments still require a digital reader to form on-site analytical devices. For truly hand-held multitarget sensing devices, Suslick's group combined an optoelectronic reader and an advanced optical sensor array to develop a series of hand-held analytical devices for on-site collection and analysis, including explosives,^114^ biomarkers,^115^ and food.^116^ Hand-held devices with reading colorimetric sensor arrays were based on digital imaging technology, such as contact image sensors (CISs), flatbed scanners, digital cameras, and cell phones. For example, CISs usually contain a linear complementary metal-oxide semiconductor sensor array for optical transduction and colorimetric data rapid collection, as shown in Fig. 7a.^117^ The detectors could receive light reflected off the object and then produce an image. Software installed in this hand-held device would process the data by comparing and analyzing each spot's color values through red, green and blue values. Based on different color values, statistical and quantitative analyses can be performed, which finally contribute to fast, sensitive, on-site sensing. For example, the quantification of urinary trimethylamine N-oxide (Fig. 7b),^118^ monitoring CO and ethylene (Fig. 7c),^119^ and examining trimethylamine from mouth and skin odor through a cell phone camera, were achieved.^115^ This portable sensing of vapors and liquids at ppb concentrations shows particular potential in point-of-care medical monitoring and diagnosis.

### 4.2 Multifunctional skin

Continuous efforts have resulted in the development of flexible/stretchable physical and bio/chemical sensors that are capable of sensing electrophysiological signals (*e.g.*, electrocardiography,^120^ electromyography^121^), physiological signals (*e.g*., pulses,^122^ temperature^123,124^), and bio/chemical parameters (*e.g.*, glucose^125^), paving the way toward the realization of multifunctional skin-like electronic systems.^27^ Therefore, we include advances in thin-film electronic systems, which enable high-performance monitoring of human activities, personal healthcare, and/or interfacing with machines. For example, inflammation-free, highly gas-permeable, ultrathin, lightweight and stretchable sensors with an Au-coated conductive nanomesh structure can be directly attached to irregular human skin for long-term effective performance. Due to the asymmetric interlocking geometry, the bioinspired e-skin is capable of real-time measurement and discrimination of normal and shear forces, with increased sensitivity, minimal hysteresis, excellent cycling stability, and response time in the millisecond range. The e-skin provided sensing feedback for controlling a robot arm during various tasks, illustrating its potential application in robotics with tactile feedback. Improved sensor characteristics in terms of spatial resolution and sensitivity, as well as the uniformity of the array, can be achieved by integrating functional sensors into active matrices. Through this approach, more delicate e-skin sensors with spatial mapping capability could be developed.

**Fig. 8:**
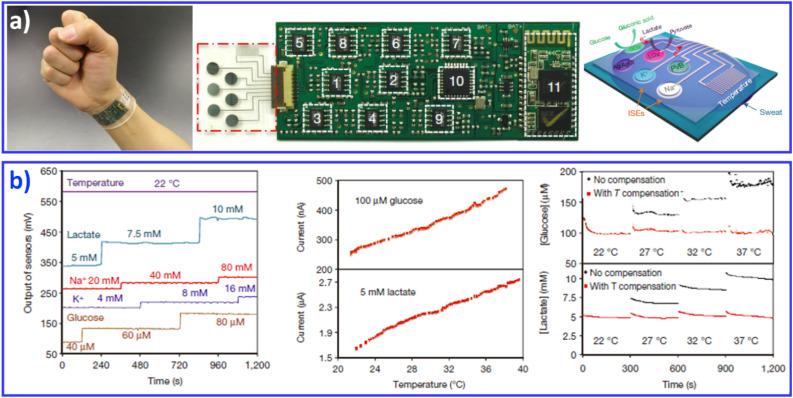
A FISA integrating a multiplexed sweat sensor array and a wireless flexible printed circuit board FPCB for the simultaneous and selective screening of a panel of biomarkers in sweat. (a) Images and schematic illustrations of the FISA. (b) System-level characterization of the sensor array. Reproduced with permission from ref. ^28^. Copyright 2016, Nature Publishing Group.

In addition to wearable sensors capable of tracking the user's physical activities and vital signs, continuous monitoring of bio/chemical parameters is of great importance to providing insight into the user's state of health at the molecular level. Measurements of human sweat, which contains rich physiological and metabolic information, could enable such insight non-invasively. Given the complexity of sweat secretions, simultaneous and multiplexed screening of target biomarkers is critical and requires full system integration to ensure the accuracy of measurements.^28,126^ Javey and co-workers developed a wearable fully integrated sensor array (FISA) for multiplexed *in situ* perspiration analysis, which simultaneously and selectively screens a panel of biomarkers in sweat, including multiple sweat metabolites (glucose and lactate), electrolytes (sodium and potassium), as well as skin temperature (Fig. 8).^28^ By integrating the FISA and a wireless flexible printed circuit board (FPCB), the wearable system integrated signal transduction, conditioning, processing and wireless transmission for the realization of a practical wearable sensor device. The system was applied to measure the detailed sweat profile of human subjects engaged in prolonged indoor and outdoor physical activities, and to make a real-time assessment of the physiological state of the subjects. The further integration of electrochemical sensors with energy modules with self-powered capability achieved noninvasive sweat monitoring, and real-time signal processing/display in a single platform.^126^

## 5. Artificial intelligence assisted perception

Realistic environments or *in vivo* samples are inherently endowed with the complexity of multiple variables and multiple features. A complete analysis will certainly yield a tremendous amount of data. In addition to performing a fast and portable response, most wearable sensing devices compromise on sensitivity or accuracy. Progress in artificial intelligence (AI) contributes convenient approaches to emancipate us from tedious work in data processing. Meyer and collaborators have endeavored to provide odor molecule recognition *via* machine-learning algorithms.^29^ They collected a psychophysical dataset from 49 individuals with profiling of 476 structurally and perceptually diverse molecules, and then quantized 4884 physicochemical features, including atom types and functional groups, as well as topological and geometrical properties. Using a large olfactory psychophysical dataset, the authors developed machine-learning algorithms to predict sensory attributes of molecules based on their chemoinformatic features. Random-forest and regularized linear models exhibit exceptionally higher performances than other common predictive model types, while tracking the observed perceptual values. This results in all predictions being reflected in a hidden test set, avoiding the pitfall of inflated correlations due to overfitting of the experimental data. The AI-assisted massive data processing and attributive correlation analysis could be run for reverse-engineering of desired perceptual profiles, to identify certain molecules from vast chemoinformatic databases and closely approach the theoretical limits of accuracy from individual variability. Recently, Tomono's group combined nanopores with machine learning to recognize four coronaviruses with high sensitivity.^127^ This simple procedure does not require a reverse transcription-polymerase chain reaction (RT-PCR), demonstrating the great potential applications of AI-assisted sensing devices.

**Fig. 9:**
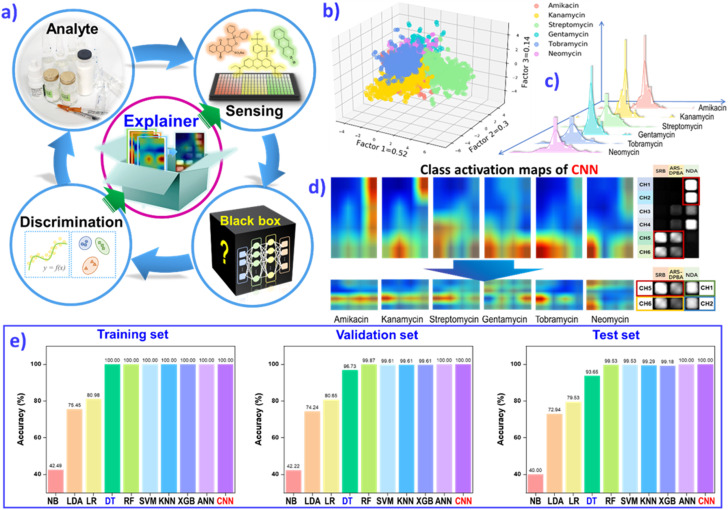
(a) The feedback mechanism of explainable deep learning (DL) guides sensor array evolution to use less material, for simplified operation or for efficient data acquisition. (b) The LDA classification plot for 6 AGs. (c) The prediction error distribution of the test set. (d) The class activation maps of AGs based on the result of the CNN model, and class activation maps of six AGs based on the result of the rebuilt CNN model after feature extraction. (e) Comparison of prediction accuracies for the AG category by employing different classification models, including NB, LDA, LR, DT, RF, SVM, KNN, XGB, MLP and CNN, on the training set, validation set and test set. The resulting assessment will guide the sensor array evolution, and approach an “end-to-end” strategy. Reproduced with permission from ref. ^129^. Copyright 2022, ACS publishing group.

The complexity and multivariate nature of biological and environmental systems are critical challenges for a high-throughput sensing methods and multianalyte identification. Deep learning (DL) algorithms present a big advantage in analyzing multidimensional data and nonlinear operation. Through efficient qualitative and quantitative contract analysis of the ten typical machine-learning algorithms, naïve-Bayes (NB), linear discriminant analysis (LDA), logistic-regression (LR), decision-tree (DT), random-forest (RF), support-vector-machine (SVM), K-nearest neighbors (KNN), extreme gradient boosting (XGBoost), multilayer perceptron (MLP) and convolutional neural network (CNN), we can conclude that the DL algorithm CNN could realize the clearer prediction of various analytes (biogenic amines, aminoglycoside antibiotics *etc.*), with 100% accuracy and multicomponent quantification (Fig. 9a).^128,129^ For example, the classification of six aminoglycoside antibiotics (AGs) was first performed through three-dimensional LDA, as shown in Fig. 9b. Then, CNN regression models were constructed to predict the concentration of AGs. The histogram of the error distribution shows many predictions with an error of ±2.5 mg L^−1^ (Fig. 9c). CNN demonstrated greater confidence and convenience than the other non-deep-learning algorithms to distinguish various analytes with clear fingerprint images.

However, for most machining-learning, especially DL, there is one puzzling cloud hanging over algorithm operations. The data-driven black boxes suffer from non-transparent inner workings. A feedback mechanism is required to explain the detailed feature extraction, and to guide the evolution of the sensor array to use less material, for simplified operation or for efficient data acquisition. Class-activation mapping assessment has been developed to explain how the CNN model assesses the importance of sensor elements and to make discrimination decisions (Fig. 9d and e). The explainable DL assisted analysis method establishes an “end-to-end” strategy, to ascertain the black-box of the DL algorithm and promote hardware design or principal optimization. The explainable AI strategy can offer a feedback mechanism to verify the design of the sensor unit, and optimize sensor devices. For future wearable platforms, the programmable sensing chip may be normalized to meet the tremendous complexity and multivariate nature of advanced health monitoring and point-of-care diagnosis, and even new scientific discoveries.

## 6. Summary and outlook

Multi-analyte discrimination has been well developed over past decades, and is still rapidly growing in terms of both sensing materials and analytical techniques. Sensor arrays have demonstrated their usefulness as mainstream tools in addressing a wide range of analytical challenges. In particular, from this review, it is apparent that micro/nanotechnology-based sensor arrays provide powerful platforms for various serials of multi-analyte discrimination. Through a combination of sensor arrays and nanotechnology, high-density sensor arrays could be miniaturized to the nanoscale. Meanwhile, new chemical and physical properties would also accompany the micro/nanometer effect, which will provide reliable sensing processes with novel sensing principles and mechanisms. For instance, facile and commercial sensor arrays combined with nanostructural substrates could replace serials of derivatives as sensors, avoiding complex rational design and fabrication processes for sensors. In addition, various sensing signals, including color, fluorescence, and resistivity, could also be adopted in a dataset for high-throughput statistical analysis. The excavation or extension of sensing signals will tremendously augment and expand the featured data matrix for discriminatory analysis with higher resolution. The advanced mathematical operation of machine-learning has been adopted to analyse extremely complex systems. Therefore, besides molecules, ions, and biomolecules as analysis targets in traditional sensor arrays, a large number of similar targets could be considered as analytes, such as transformation positions, human motions, human facial expressions, and human olfactory perception. These novel targets would greatly extend the application areas of sensor arrays in differential detection.

Although much progress has been achieved in recent years, great challenges still remain in micro–nanostructural sensor arrays with miniaturization, integration and high sensitivity, especially customizable features for the target-oriented discrimination of a broad spectrum of multiple analytes. Based on the bottom layer of the response data matrix and cross-reactive operation, three promoted strategies could be summarized: (1) creating material diversity, (2) designing the organization of a sensor array structure, and (3) integrating multifunctional devices. The excellent optical or electronical properties on nanoparticles, nanofibers or nanomatrices contribute significant sensitivity and selectivity for traditional or novel sensing processes. Ingenious and well-organized or assembled materials to design structures, can endow them with particular response features for ergonomic analysis. Device integration can collect various functions to match practical requirements. With the rational design of micro–nanostructural sensor arrays, responsive materials after binding with target analytes could be introduced into sensor arrays as flat forms to achieve chemical and physical property tunability. The adoption of deep-learning (DL) algorithms will contribute a big advantage to analysing nonlinear, multidimensional and massive data. But most DL models are confronted with a “black-box” dilemma: the non-transparency of neural networks. By opening the “black-box” and pointing out the connection between input and output, the explanatory AI analysis method can establish an end-to-end strategy. Ascertaining the activation and decision-making mechanisms of the AI algorithm can help to assess the efficiency of sensor units, which will promote hardware design, principle optimization, even new scientific discoveries. In the end, there are still big challenges to the performance of micro–nanostructural sensor arrays in practical applications, such as the lack of a corresponding platform, signal singularity, the inconvenience of information extraction, integration with wearable devices, and an incomplete system. Especially for on-site application, a sensor array's long-term durability and mechanical stability still need improvement. On the other hand, since sensor arrays and multi-analyte discrimination have been demonstrated, their applications in areas related to the monitoring of human health, such as blood, urine, sweat, and motion, the construction of sensor arrays on wearable devices with biocompatibility and bioactive functions should be taken into consideration to adapt the tendency of wearable or implantable intelligent devices.

## Author contributions

The manuscript was written through contributions of all authors. All authors have given approval to the final version of the manuscript.

## Conflicts of interest

There are no conflicts to declare.

## Supplementary Material
